# Contrast-enhanced mammography for the assessment of screening recalls: a two-centre study

**DOI:** 10.1007/s00330-022-08868-3

**Published:** 2022-06-01

**Authors:** Andrea Cozzi, Simone Schiaffino, Marianna Fanizza, Veronica Magni, Laura Menicagli, Cristian Giuseppe Monaco, Adrienn Benedek, Diana Spinelli, Giovanni Di Leo, Giuseppe Di Giulio, Francesco Sardanelli

**Affiliations:** 1 Department of Biomedical Sciences for Health, Università degli Studi di Milano, Via Luigi Mangiagalli 31, 20133 Milano, Italy; 2 Unit of Radiology, IRCCS Policlinico San Donato, Via Rodolfo Morandi 30, 20097 San Donato Milanese, Italy; 3 Department of Breast Radiology, Fondazione IRCCS Policlinico San Matteo, Viale Camillo Golgi 19, 27100 Pavia, Italy

**Keywords:** Breast neoplasms, Biopsy, needle, Ductal carcinoma in situ, Mammography, contrast-enhanced, Mass screening

## Abstract

**Objectives:**

To evaluate the potential of contrast-enhanced mammography (CEM) for reducing the biopsy rate of screening recalls.

**Methods:**

Recalled women were prospectively enrolled to undergo CEM alongside standard assessment (SA) through additional views, tomosynthesis, and/or ultrasound. Exclusion criteria were symptoms, implants, allergy to contrast agents, renal failure, and pregnancy. SA and CEM were independently evaluated by one of six radiologists, who recommended biopsy or 2-year follow-up. Biopsy rates according to SA or recombined CEM (rCEM) were compared with the McNemar’s test. Diagnostic performance was calculated considering lesions with available final histopathology.

**Results:**

Between January 2019 and July 2021, 220 women were enrolled, 207 of them (median age 56.6 years) with 225 suspicious findings analysed. Three of 207 patients (1.4%) developed mild self-limiting adverse reactions to iodinated contrast agent. Overall, 135/225 findings were referred for biopsy, 90/225 by both SA and rCEM, 41/225 by SA alone and 4/225 by rCEM alone (2/4 being one DCIS and one invasive carcinoma). The rCEM biopsy rate (94/225, 41.8%, 95% CI 35.5–48.3%) was 16.4% lower (*p* < 0.001) than the SA biopsy rate (131/225, 58.2%, 95% CI 51.7–64.5%). Considering the 124/135 biopsies with final histopathology (44 benign, 80 malignant), rCEM showed a 93.8% sensitivity (95% CI 86.2–97.3%) and a 65.9% specificity (95% CI 51.1–78.1%), all 5 false negatives being ductal carcinoma in situ detectable as suspicious calcifications on low-energy images.

**Conclusions:**

Compared to SA, the rCEM-based work-up would have avoided biopsy for 37/225 (16.4%) suspicious findings. Including low-energy images in interpretation provided optimal overall CEM sensitivity.

**Key Points:**

• *The work-up of suspicious findings detected at mammographic breast cancer screening still leads to a high rate of unnecessary biopsies, involving between 2 and 6% of screened women.*

• *In 207 recalled women with 225 suspicious findings, recombined images of contrast-enhanced mammography (CEM) showed a 93.8% sensitivity and a 65.9% specificity, all 5 false negatives being ductal carcinoma in situ detectable on low-energy images as suspicious calcifications.*

• *CEM could represent an easily available one-stop shop option for the morphofunctional assessment of screening recalls, potentially reducing the biopsy rate by 16.4%.*

**Supplementary Information:**

The online version contains supplementary material available at 10.1007/s00330-022-08868-3.

## Introduction

While the benefits of mammographic screening outweigh its harms [1–4], various issues of the whole screening process are still unresolved [3]. Alongside a strong drive towards personalisation of screening strategies [5], research efforts are targeting a major drawback of mammographic screening, i.e. false positive recalls [3]. Indeed, even the current multi-layered imaging assessment still implies that women undergoing screening mammography have an estimated cumulative risk of undergoing a biopsy with a final benign outcome ranging between 2 and 6% [3, 6]. This figure is mirrored by the constantly high proportion of benign lesions (between 44 and 73%) reported in large-scale biopsy series [7–10].

Currently, the most employed assessment modalities—such as additional mammographic views, digital breast tomosynthesis, and ultrasound—rely exclusively on a morphologic appraisal of suspicious findings. Conversely, imaging techniques able to provide morphologic and functional information may foster a decrease in biopsy rates, i.e. an increase in the positive predictive value (PPV) of work-up examinations. This notion rests on the biological bases of functional assessment through contrast-enhanced examinations: tumour neoangiogenesis, resulting in leaky vessels that allow the entry of contrast agents into the interstitium, is a predominant feature of invasive cancers and more aggressive lesions [11, 12].

Among morpho-functional breast imaging techniques, contrast-enhanced mammography (CEM) could be better suited [13–15] than contrast-enhanced breast magnetic resonance imaging (CE-MRI) [16] for the work-up of screen-detected suspicious findings, as the latter has considerable contraindications, cost-related pitfalls, and may be suboptimal in assessing calcifications [17]. The potential of CEM has been highlighted also by a recent meta-analysis [18], where CEM had a 92% sensitivity and an 84% specificity when applied on mammography-detected suspicious findings.

CEM consists in a pair of mammograms (one low-energy, one high-energy) sequentially acquired after contrast agent administration and then recombined to minimize the appearance of unenhancing breast tissue, making enhanced areas recognizable [19]. Moreover, save from contrast administration, CEM is similar in workflow and time to a standard 4-view mammography or tomosynthesis [20], thus being much more tolerated, affordable, and available than CE-MRI [21–24].

The aim of this study was therefore to assess the potential of CEM for curtailing the biopsy rate in a prospectively enrolled population of women recalled for assessment of suspicious findings at screening mammography.

## Methods

### Study design and population

Approval for this bicentric prospective study was obtained by the Ethics Committee of IRCCS Ospedale San Raffaele, Milan, Italy (protocol code CESM; approved May 10th, 2018) and by the Ethics Committee of Fondazione IRCCS Policlinico San Matteo, Pavia, Italy (protocol code P-20190076950, approved September 25th, 2019).

Enrolment in this study was proposed to all women aged 40–80 years referred to the Radiology Unit of IRCCS Policlinico San Donato, San Donato Milanese, Milan, Italy (Centre 1), or to the Department of Breast Radiology of Fondazione IRCCS Policlinico San Matteo, Pavia, Italy (Centre 2), for the work-up of suspicious findings detected at screening mammography (the structure and logistics of the local screening program being described in the Supplementary Material), between January 25th, 2019, and July 29th, 2021. Exclusion criteria were as follows: breast symptoms suspicious for breast cancer; pregnancy; presence of breast implants; allergy to iodinated contrast agents; renal failure (estimated glomerular filtration rate < 30 mL/min × 1.73 m^2^).

At both centres, standard assessment (SA) of suspicious findings was performed with additional mammographic views including mammographic magnification and/or spot compression, ultrasound, or digital breast tomosynthesis, according to the characteristics of each investigated suspicious finding.

Eligible women willing to provide informed consent entered this study and, after collection of personal data (age, height, weight, menstrual cycle status), underwent CEM immediately after SA, as depicted in the protocol flowchart (Fig. E1).

### Image acquisition and analysis

All CEM examinations were performed on a Senographe Pristina mammography system (GE Healthcare) at both centres. The following imaging protocol was used at both centres: 2 min before the first image acquisition, a 1.5 mL/kg dose of a non-ionic, monomeric, low-osmolar contrast agent (Iohexol 350 mgI/mL; GE Healthcare) was administered intravenously with an automated injector at a 2 mL/s flow rate, followed by a 30 mL saline flush. Then, standard mediolateral oblique and craniocaudal views were obtained in a maximum timeframe of 10 min, following the acquisition sequence commonly applied for diagnostic mammography at each centre [20]. All examinations times and the occurrence of any adverse reaction were recorded.

At each centre, two readers were involved in the interpretation of each patient’s examinations. The reader who performed the routine SA had no access to CEM; vice versa, CEM was independently interpreted by another reader, who was blinded to the results of the SA but aware of the mammographic findings that prompted the recall and had unrestricted access to the original mammographic images. Overall, six readers with a breast imaging experience ranging 6–30 years were involved in the interpretation process in the two centres.

SA results were categorised according to the BI-RADS classification [25] and women were either referred to biopsy or entered a 2-year follow-up with routine screening mammography and/or breast ultrasound. Conversely, since the reader interpreting CEM had access to the original mammographic images and CEM low-energy images are technically equivalent to a standard mammographic exam [26, 27] in providing a morphologic evaluation of the suspicious findings, CEM interpretation was focused on the recombined images (rCEM), in order to investigate the added value of the functional information provided by these contrast-enhanced images. On the basis of rCEM readings, the reader assessing CEM defined negative findings (i.e. those not needing a biopsy according to rCEM evaluation) and positive findings (those warranting a biopsy referral according to rCEM evaluation). If the reader interpreting CEM identified suspicious lesions different from those that prompted the recall and needing a dedicated work-up, the information was disclosed to the colleague performing SA and the work-up of these additional abnormalities was immediately performed according to the clinical practice currently used for additional findings at breast CE-MRI (targeted ultrasound, additional mammograms/tomosynthesis views, image-guided biopsy). Of note, as this design aims to evaluate the potential of rCEM to reduce the biopsy rate, CEM results could only be used to refer women to biopsy for suspicious findings that were not detectable at SA: biopsies recommended by SA were always performed, even with negative rCEM results.

### Statistical analysis

The primary endpoint of this study was the potential rCEM biopsy rate, to be compared with the effectively performed SA biopsy rate, respectively calculated as

$$ rCEM\ biopsy\ rate=\frac{suspicious\ findings\ referred\ to\ biopsy\ according\ to\ rCEM\ }{total\ suspicious\ findings\ in\ enrolled\ women} $$

and

$$ SA\  biopsy\ rate=\frac{suspicious\ findings\ referred\ to\ biopsy\ according\ to\  SA}{total\ suspicious\ findings\ in\ enrolled\ women} $$

Secondary endpoints were as follows: (1) the number of adverse reactions to iodinated contrast agents (classified according to the 2021 American College of Radiology Manual [28]), and (2) SA and rCEM diagnostic performance, taking histopathology or 2-year follow-up as reference standard, considering in particular the number of detected and missed malignancies and, among them, of ductal carcinomas in situ (DCIS). For the latter secondary endpoint, we here present a subanalysis restricted to cases with available final histopathology reports, since the follow-up period is still ongoing.

Considering the presence of experienced breast radiologists at both centres and based on previous internal reviews of biopsy rates, we preliminarily assumed that women enrolled in this study would have a SA biopsy rate of about 50% and that rCEM could lead to about a 20% reduction in biopsy rate. We therefore calculated the sample size under the hypothesis of clinical superiority (i.e. of reducing the biopsy rate), assuming an 80% statistical power and a 5% α error. Under these assumptions, 197 women needed to be enrolled.

The Shapiro-Wilk test was used to perform distribution analysis. Consequentially, normal distributions were reported using mean ± standard deviation and non-normal distributions were reported as median with their interquartile range (IQR). The paired data comparison for the primary endpoint was performed with the McNemar’s test (*p* values < 0.05 considered statistically significant), while rates and diagnostic performance metrics for the secondary endpoints were determined along with their 95% confidence intervals (CIs). All analyses were performed with STATA, version MP 16.1 (StataCorp LLC).

## Results

Between January 25, 2019, and July 29, 2021, 220 women were enrolled in this study, 122 at Centre 1 and 98 at Centre 2. CEM proved unfeasible in 3 of these 220 women (1.4%) because of contrast extravasation, while 10 other women were excluded from analysis after enrolment due to screening failure of exclusion criteria. The remaining 207 women who underwent both SA and CEM were included in the analysis: they had a median age of 56.6 years (IQR 50.1–65.3 years), 140/207 (67.6%) had already entered menopause, and 26/207 (12.6%) reported a family history of breast or ovarian cancer, no woman declaring to be a carrier of a genetic mutation increasing breast cancer risk. Out of 207 patients, 3 (1.4%) developed mild self-limiting adverse reactions to iodinated contrast agents, without the need of any medical intervention. The median CEM examination time was 4 min and 46 s (286 s, IQR 262–318 s).

The SA was prompted by a single suspicious finding in 191/207 women (92.3%), while in the remaining 16/207 women (7.7%) SA detected 2 suspicious findings (ipsilateral in 12 women, contralateral in 4 women). Of these 223 suspicious findings, 214 (95.9%) were already detectable on baseline mammography, 3/223 (1.4%) were suspicious axillary lymph nodes detected by ultrasonography, and the remaining 6/223 (2.7%) were inconclusive mammographic findings that were confirmed as suspicious by ultrasonography. Moreover, in 2 women (1.0%) rCEM identified an additional suspicious finding (both of them in the breast contralateral to the suspicious finding that prompted the recall).

**Fig. 1:**
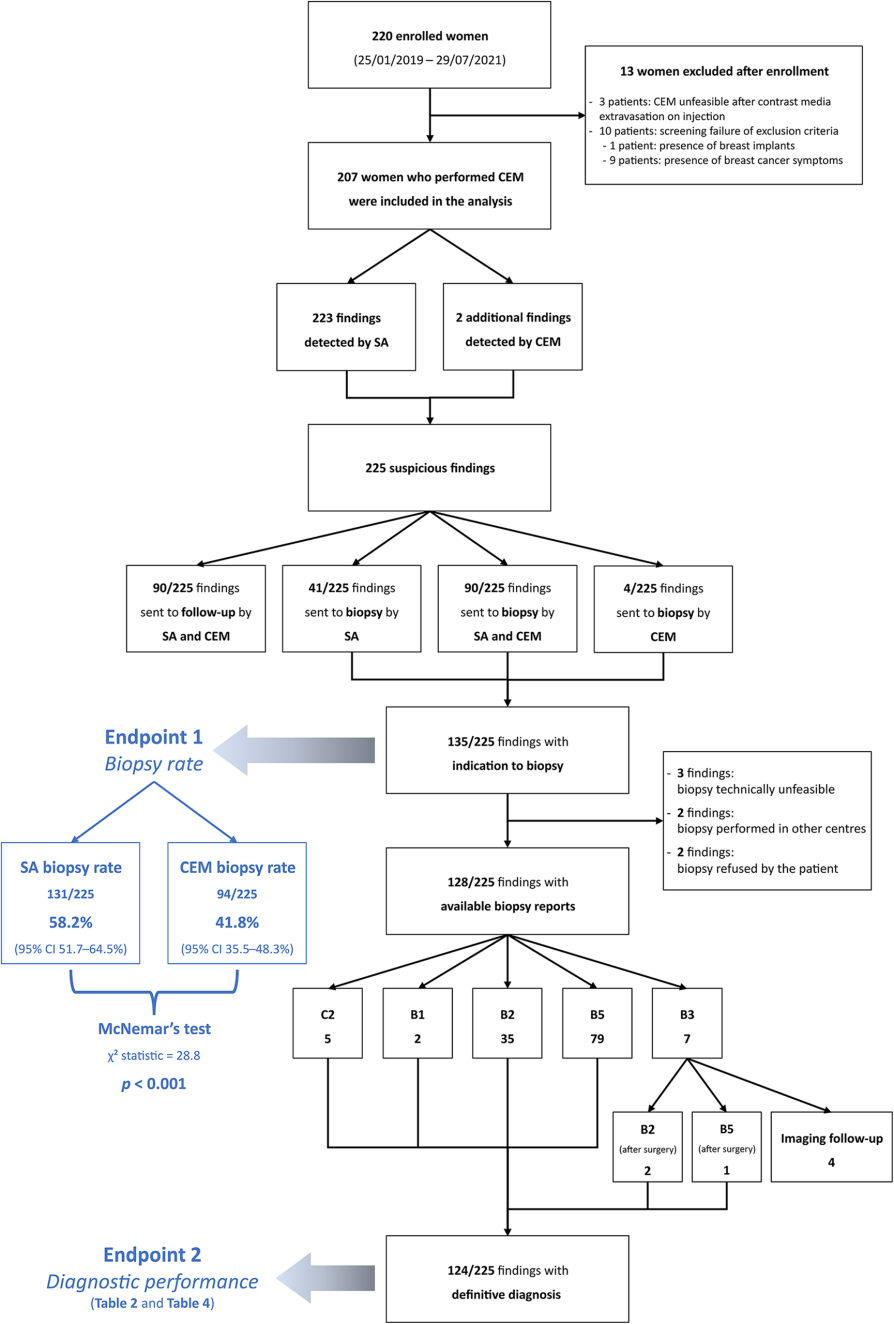
Study phases and endpoint analyses

As detailed in the study flowchart (Fig. 1), 225 suspicious findings were ultimately analysed for the assessment of the primary endpoint (Tables E1–E4): 131/225 were referred to biopsy by SA, for a SA biopsy rate of 58.2% (95% CI 51.7–64.5%), while 94/225 were referred to biopsy by rCEM, for a rCEM biopsy rate of 41.8% (95% CI 35.5–48.3%). Therefore, information from rCEM images would have engendered a 16.4% reduction in the biopsy rate, from 58.2 to 41.8% (*p* < 0.001). More specifically, SA and rCEM agreed on referring to biopsy 90/225 (40.0%) suspicious findings and agreed on sending to follow-up 90/225 (40.0%) suspicious findings. Conversely, rCEM would have spared the biopsy prompted by SA in 41/225 cases (18.2%) and effectively recommended biopsy for 4 findings (1.8%): 2 would have been sent to follow-up according to the SA, and 2 were rCEM-only detected findings. Thus, a biopsy was recommended either by SA or by rCEM for 135 suspicious findings. For 3 of them the procedure proved unfeasible, 2 other women elected to perform the recommended biopsy in other centres and were lost at follow-up, and 2 women—for whom CEM recommended a biopsy in contrast to the follow-up referral recommended by SA—refused to undergo the procedure.

Ultimately, 128 biopsies were performed at the two study centres, 75/128 (58.6%) under ultrasound guidance and 53/128 (41.4%) under stereotactic guidance. Overall, all 53 stereotactic-guided biopsies and 2 of the ultrasound-guided biopsies were performed as vacuum-assisted biopsies, while among the 73 remaining ultrasound-guided biopsies 68 (93.1%) were core-needle biopsies and 5 (6.9%) were fine-needle sampling. As detailed in Table 1, 42/128 biopsies had a benign result (32.8%) and 79/128 resulted in a diagnosis of malignancy (61.7%): DCIS accounted for 31.6% of malignancies (25/79). The remaining 7/128 biopsies (5.5%) had a B3 result: 4 cases were sent to imaging follow-up and were excluded from secondary endpoint analyses, while the other 3 were referred for surgery, 2 being downgraded to B2 lesions at surgical histopathology and one upgraded to a B5b lesion.

**Table 1:** Results of the 128 percutaneous breast biopsies performed in the two study centres. 126 biopsies were performed after recommendation by standard assessment (89 with concurrent referral by contrast-enhanced mammography) and two were solely prompted by findings at contrast-enhanced mammography

Biopsy classification	Histological type	Number	%
B1	Normal parenchyma	2	1.6%
B2	Acute mastitis	1	0.8%
	Adenosis	6	4.7%
	Adenosis with fibrocystic changes	3	2.3%
	Adenosis with fibrosis	3	2.3%
	Adenosis with usual ductal hyperplasia	4	3.1%
	Apocrine metaplasia	3	2.3%
	Columnar cell hyperplasia without atypia	2	1.6%
	Fibroadenoma	5	3.9%
	Fibrocystic changes	7	5.4%
	Inflammatory changes	1	0.8%
C2	Normal cytology	5	3.9%
B3 referred for surgery	Atypical ductal hyperplasia^a^	2	1.6%
	Flat epithelial atypia ^b^	1	0.8%
B3 referred for imaging follow-up	Columnar cell hyperplasia with atypia	1	0.8%
	Flat epithelial atypia	2	1.6%
	Flat epithelial atypia and atypical ductal hyperplasia	1	0.8%
B5	DCIS grade 1–grade 2	1	0.8%
	DCIS grade 2	10	7.8%
	DCIS grade 3	8	6.3%
	DCIS grade 2 with associated microinvasion	3	2.3%
	DCIS grade 3 with associated microinvasion	3	2.3%
	IC NST grade 1	7	5.4%
	IC NST grade 2	21	16.4%
	IC NST grade 3	7	5.4%
	IC NST grade 1 with associated DCIS grade 1	1	0.8%
	IC NST grade 1 with associated DCIS grade 2	1	0.8%
	IC NST grade 2 with associated DCIS grade 2	2	1.6%
	IC NST grade 2 with associated DCIS grade 3	3	2.3%
	IC NST grade 3 with associated DCIS grade 3	1	0.8%
	Invasive lobular carcinoma	2	1.6%
	Invasive lobular carcinoma with associated LCIS	2	1.6%
	Invasive papillary carcinoma	2	1.6%
	Medullary carcinoma	1	0.8%
	Metastatic lymph node	4	3.1%

*DCIS* ductal carcinoma in situ, *IC* invasive carcinoma, *NST* no special type, *LCIS* lobular carcinoma in situ

^a^Both cases downgraded to B2 at surgical histopathology

^b^Upgraded to B5 (invasive carcinoma of no special type, grade 2) at surgical histopathology

**Fig. 2:**
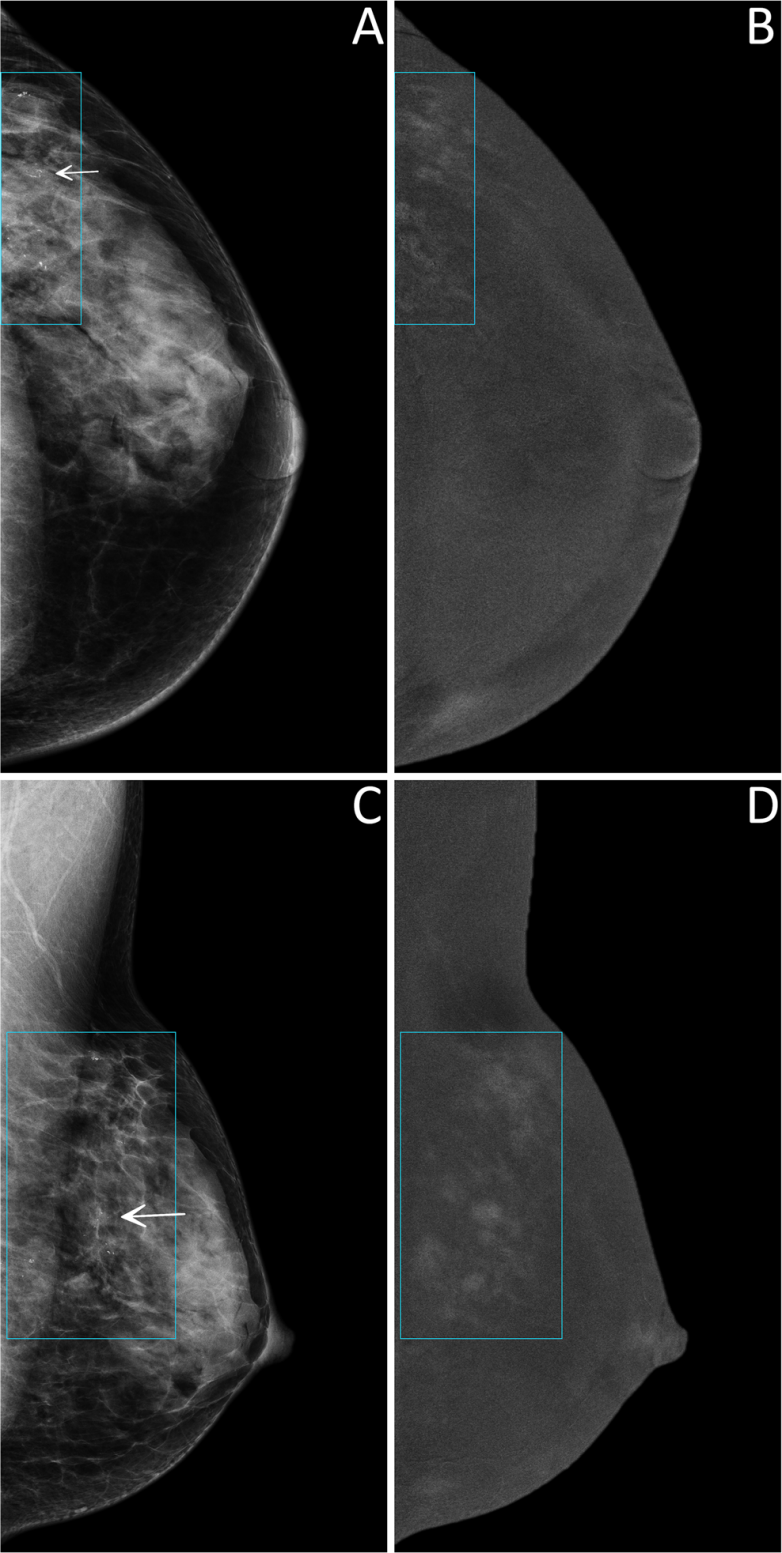
True positive case at contrast-enhanced mammography. A 53-year-old woman was recalled for suspicious calcifications in the left breast. An ultrasound-guided core needle biopsy was performed, resulting in a diagnosis of grade 2 ductal carcinoma in situ. Low-energy images (panels **A** and **C**) show multiple groups of pleomorphic calcifications in the left upper-outer quadrant (white arrows in light blue rectangles). Recombined images (panels **B** and **D**, light blue rectangles) revealed an area of non-mass enhancement involving the whole upper-outer quadrant

**Fig. 3:**
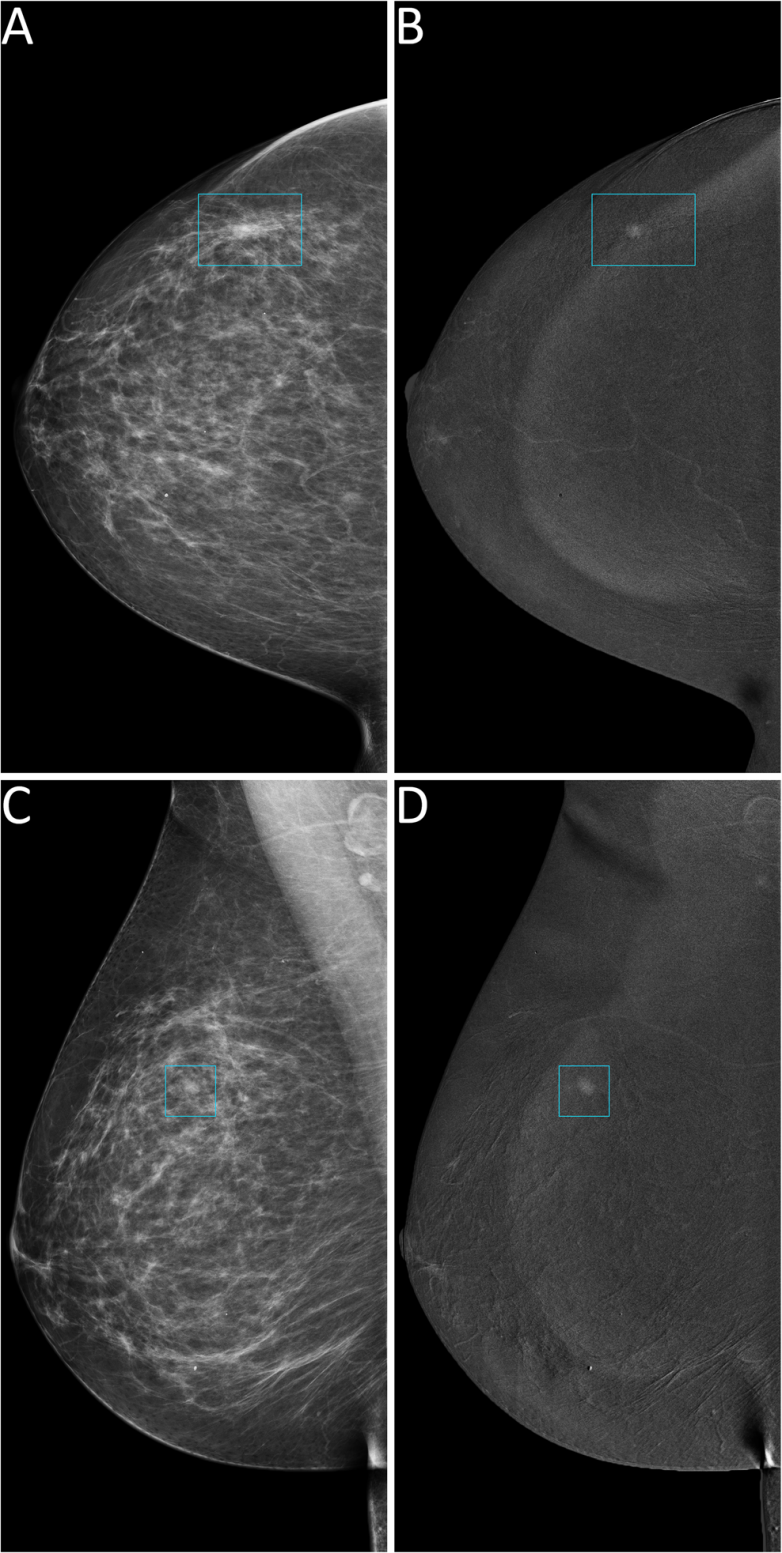
False positive case at contrast-enhanced mammography. A 69-year-old woman was recalled for a suspicious finding in the right breast, subsequently diagnosed as adenosis. Low-energy images (panels **A** and **C**) show a small opacity in the right upper-outer quadrant (light blue rectangles) with a correlated sub-centimetric enhancement focus on the recombined images (panels **B** and **D**, light blue rectangles)

**Fig. 4:**
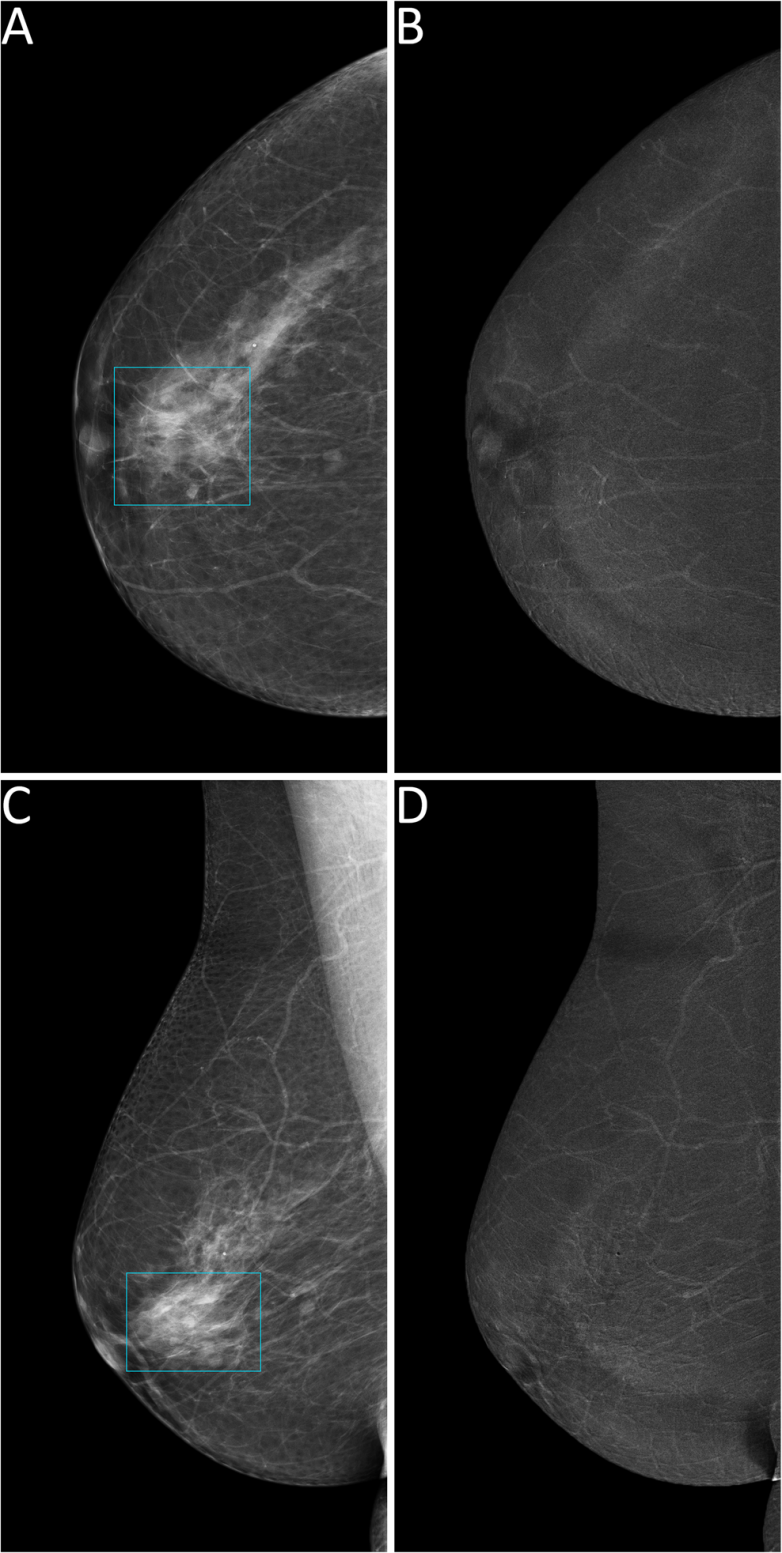
True negative case at contrast-enhanced mammography. A 58-year-old woman was recalled for a suspicious retroareolar irregular opacity in the right breast (panel **A** and **C**, light blue rectangles). An ultrasound-guided core needle biopsy was performed, leading to a diagnosis of apocrine metaplasia. The absence of enhancement foci on recombined images (panels **B** and **D**) would have oriented the referral to follow-up

**Fig. 5:**
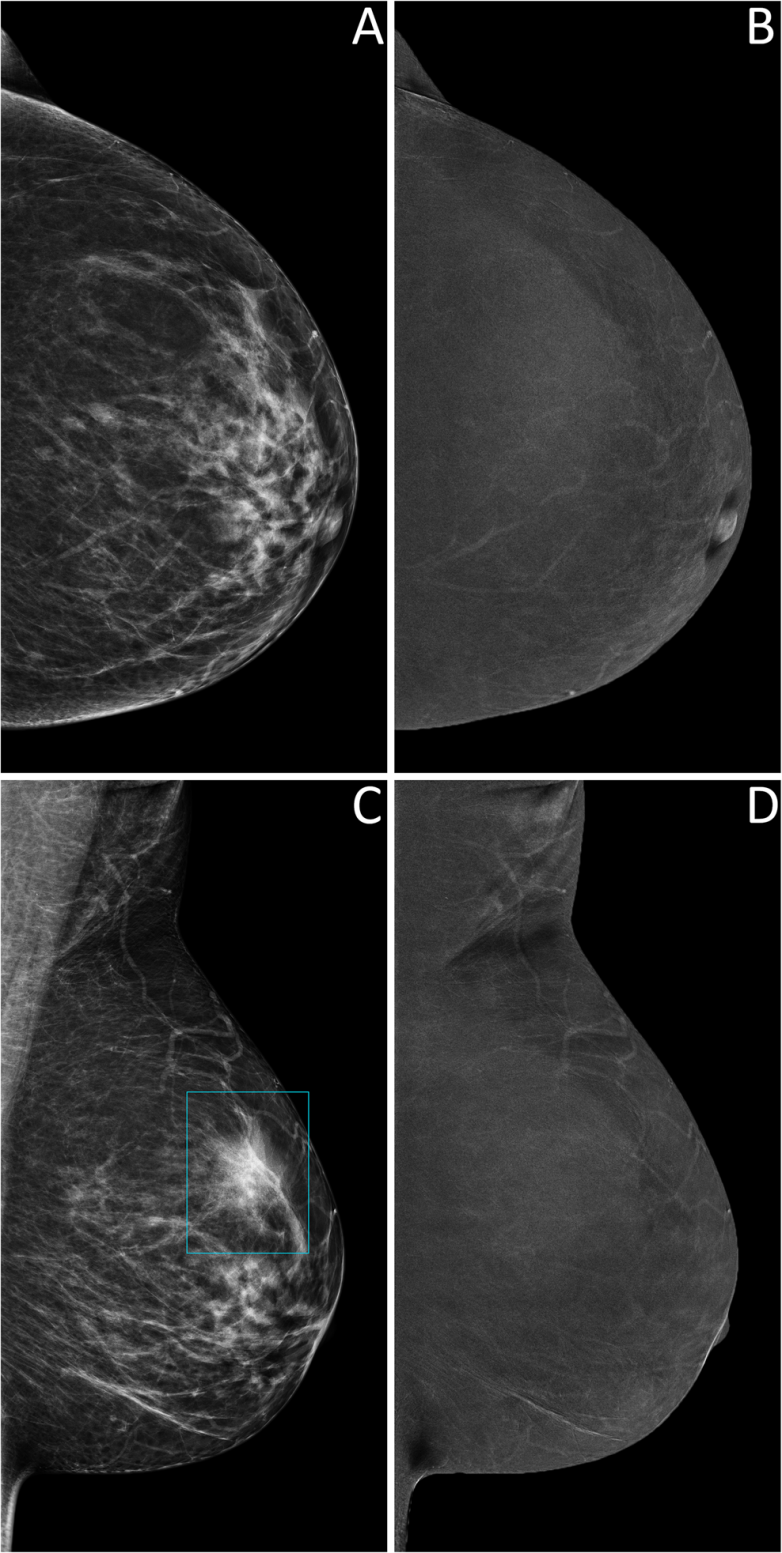
True negative case at contrast-enhanced mammography. A 49-year-old woman was recalled for a suspicious asymmetry in the upper quadrants of the left breast, not observable on the craniocaudal view (low-energy image, panel **A**) but definitely noticeable on the mediolateral oblique view (low-energy image, panel **C**, light blue rectangle). Standard assessment referred this finding to ultrasound-guided core needle biopsy, leading to a diagnosis of fibrosis. Conversely, the absence of enhancement in recombined images, both on the whole craniocaudal view (panel **B**) and in correspondence of the suspicious area on the mediolateral oblique view (panel **D**) would have oriented the work-up to a normal result with referral to re-screening at a 2-year interval

**Fig. 6:**
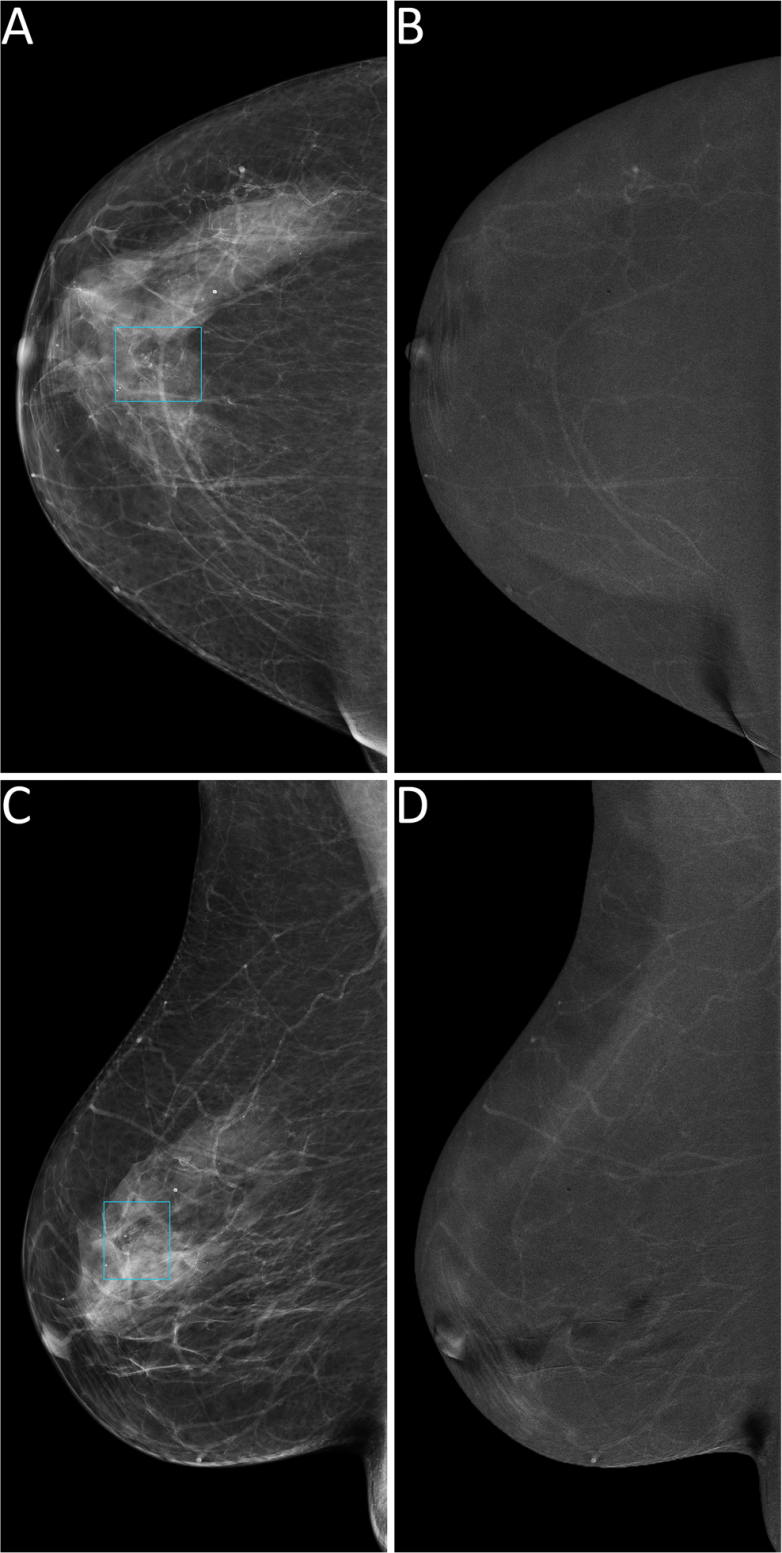
False negative case at contrast-enhanced mammography. A 67-year-old woman was recalled for a suspicious group of pleomorphic calcifications in the in the upper quadrants of the right breast, subsequently diagnosed as a grade 2 ductal carcinoma in situ, clearly visible on low-energy contrast-enhanced mammography images (panels **A** and **C**, light blue rectangles) but not associated with any enhancement on recombined images (panels **B** and **D**)

Thus, 124 lesions (44 benign and 80 malignant, 25 of which DCIS) had an available final histopathology report and were considered for the evaluation of the secondary endpoints related to diagnostic performance. Among the 122/124 lesions sent to biopsy by SA, 44 (36.1%) proved benign at histopathology, while the remaining 78 (63.9%) were classified as malignant, 24 of them being DCIS. The 2/124 suspicious findings that were not detected by SA but had a biopsy prompted by rCEM also resulted to be B5 lesions (one grade 2 DCIS and one invasive carcinoma of no special type). The sensitivity of SA (Table 2) was therefore 97.5% (95% CI 91.3–99.3%), with a PPV of 63.9% (95% CI 55.1–71.9%). Among the 90 suspicious findings sent to biopsy according to the information coming from rCEM images, 75/90 (83.3%, 20/90 DCIS) were malignant lesions (true positives, Fig. 2 and Fig. E2), while the remaining 15/90 (16.7%) were benign lesions (false positives, Fig. 3) Conversely, among the 34 biopsies with final reports that would have been spared by the evaluation of rCEM images (Table 3), histopathology revealed 29 benign (true negatives, Figs. 4 and 5) and 5 malignant lesions (false negatives, Fig. 6). Of note, all 5 were pure DCIS, i.e. without microinvasion (3 grade 2 and 2 grade 3): while none of them exhibited suspicious contrast enhancement on rCEM images, all were detectable on low-energy CEM images due to the presence of suspicious calcifications. Thus, while rCEM sensitivity (Table 4) was 93.8% (95% CI 86.2–97.3%), with a 65.9% specificity (95% CI 51.1–78.1%) and an 83.3% PPV (95% CI 74.3–89.6%), a combined reporting of rCEM images and low-energy images (focused on suspicious calcifications) to guide biopsy referral would have increased sensitivity to 100% (95% CI 95.4−100.0%).

**Table 2:** Diagnostic performance indexes for the standard assessment, calculated on 124 lesions with available final histopathology results

		Histopathology		
		Malignant	Benign	
Standard assessment	Positive	78	44	PPV 63.9% (55.1–71.9%)
	Negative	2^a^	0^b^	NPV —
		Sensitivity 97.5% (91.3–99.3%)	Specificity —	Accuracy —

Numbers in parentheses indicate 95% confidence intervals. Dashes indicate that the corresponding diagnostic performance index was not calculated, being influenced by the absence of true negative lesions in this preliminary analysis of diagnostic performance focused on lesions referred for biopsy by any of the two imaging modalities

*PPV* positive predictive value, *NPV* negative predictive value

^a^Biopsy prompted by findings at contrast-enhanced mammography

^b^In temporary absence of follow-up data. No lesions without a referral from either standard assessment or contrast-enhanced mammography underwent a biopsy

**Table 3:** Final histopathology results of the 34 percutaneous breast biopsies that were effectively performed but would have been spared by information coming from recombined contrast-enhanced mammography images

Biopsy classification	Histological type	Number	%
B2	Acute mastitis	1	2.9%
	Adenosis	4	11.8%
	Adenosis with fibrocystic changes	3	8.8%
	Adenosis with fibrosis	3	8.8%
	Adenosis with usual ductal hyperplasia	1	2.9%
	Apocrine metaplasia	3	8.8%
	Columnar cell hyperplasia without atypia	2	5.9%
	Fibroadenoma	2	5.9%
	Fibrocystic changes	5	14.8%
C2	Normal cytology	3	8.8%
B3 referred for surgery	Atypical ductal hyperplasia^a^	2	5.9%
B5	DCIS grade 2	3	8.8%
	DCIS grade 3	2	5.9%

Contrast-enhanced mammography would have spared 7 other biopsies that were indicated by standard assessment: in three cases biopsy proved unfeasible, in one case the patient elected to perform the biopsy in another centre and was lost at follow-up, and the remaining three cases were B3 lesions (two cases flat epithelial atypia and one case of columnar cell hyperplasia with atypia) sent to imaging follow-up

*DCIS* ductal carcinoma in situ

^a^Both cases downgraded to B2 at surgical histopathology

**Table 4:** Diagnostic performance indexes for the recombined contrast-enhanced mammography images, calculated on 124 lesions with available final histopathology results

		Histopathology		
		Malignant	Benign	
rCEM	Positive	75	15	PPV 83.3% (74.3–89.6%)
	Negative	5	29	NPV 85.3% (69.9–93.6%)
		Sensitivity 93.8% (86.2–97.3%)	Specificity 65.9% (51.1–78.1%)	Accuracy 83.9% (76.4–89.3%)

Numbers in parentheses indicate 95% confidence intervals

*rCEM* recombined images from contrast-enhanced mammography, *PPV* positive predictive value, *NPV* negative predictive value

## Discussion

Since the early days of CEM implementation, its use in the evaluation of abnormalities detected at screening mammography has been one of the most reported applications [14, 15]. Albeit with some caveats related to the contrast uptake of benign lesions [14, 15] and to equivocal enhancement conspicuity associated with calcifications clusters [29–31], retrospective studies have highlighted the potential of CEM to increase the PPV of the work-up process without compromising cancer detection [31–35]. We investigated this issue in a prospective setting, assessing the diagnostic gain granted by contrast-enhanced (rCEM) images, since low-energy CEM images—equivalent to standard mammograms [26, 27]—are also available in the SA process used as a comparator.

We observed a potential 16.4% net reduction of the biopsy rate that could be obtained by rCEM in the overall cohort of 225 suspicious findings, accompanied, in a subanalysis on 124 findings with final diagnosis, by a 19.4% PPV increase, in accordance with the multireader retrospective study by Zuley et al [35] on 60 BI-RADS 4 masses referred for biopsy. While their higher negative predictive value (98.3% versus our 85.3%) was likely prompted also by their exclusion of calcifications, we found similar, even though slightly higher, sensitivity (93.8% versus 90.3%) and specificity (65.9% versus 61.0%). Of note, we should consider that our specificity was negatively influenced by the exclusion of lesions referred for follow-up and will be recalculated after follow-up completion.

The biopsy increase solely attributable to CEM, i.e. the number of CEM-referred biopsies of suspicious findings that would have been sent to follow-up by SA plus the number of additional suspicious lesions detected by CEM but missed by screening mammography and SA, was 4/225 (1.8%). While the component of additional CEM-only findings (2/225, 0.9%) is of course lower than the 7.7% rate presented by Houben et al [34] in a study where screening mammography was the comparator instead of SA, we highlight that both cases in which the patient accepted to undergo the biopsy solely prompted by CEM were diagnosed as malignant lesions (one invasive carcinoma of no special type, one grade 2 DCIS), with a 100% PPV.

Importantly, DCIS presenting as calcifications clusters without associated contrast enhancement or with extremely faint enhancement were altogether responsible for the 6.2% drop in sensitivity of rCEM compared to the virtual 100% sensitivity of a combined reporting of low-energy images focused on suspicious calcifications and rCEM images, thus still supporting a direct biopsy referral of suspicious calcifications on the basis of their appearance on standard mammography or low-energy CEM images [31]. Without venturing in considerations about potential DCIS overdiagnosis [36], we however highlight that all were pure DCIS, without any microinvasion foci (3 intermediate grade, 2 high grade). As already reported [29–31], the negative predictive value of rCEM images for suspicious calcifications remains to be ascertained and, in our opinion, only large-scale dedicated studies will allow to solve this issue, especially also addressing DCIS overdiagnosis. Options in this direction involve the identification of characteristic enhancement patterns for cancers of low biological relevance [37] and the application of artificial intelligence–driven radiomic analysis [38]. The latter could be particularly useful considering how interpretation thresholds are influenced by the more equivocal visual conspicuity of lesion enhancement in rCEM images than in CE-MRI, compared to standard background parenchymal enhancement. In addition, only 3/207 patients (1.4%) developed mild self-limiting adverse reactions to iodinated contrast agent, confirming the CEM safety profile already reported in a meta-analysis [20].

Limitations of this study include—first—the only potential nature of the biopsy reduction we described and the non-randomised design: these characteristics prevented a clinical comparison of the SA and CEM-based work-up, also including patients’ preferences and cost-effectiveness, as will be done by the RACER trial [39]. Second, as already discussed for suspicious calcifications resulting in rCEM false negatives, our study design also factually oriented the analysis towards an appraisal of the contribution of rCEM information rather than of the “whole” CEM examination (low-energy and rCEM images). Finally, the ongoing follow-up period prevented us from exploring secondary endpoints related to diagnostic performance in the whole cohort, such as the correlation of imaging features with histopathology.

In conclusion, our study showed how a rCEM-based assessment of women recalled at first-level screening mammography is able to potentially engender a 16.4% reduction in biopsy rates compared to SA, maintaining high sensitivity (93.8%) with false negatives represented only by DCIS clearly detectable on low-energy CEM images. Coupled with the absence of moderate and severe adverse reactions to contrast agent, these data further highlight the role of CEM for the assessment of suspicious findings detected at screening mammography, avoiding a sizable number of unnecessary biopsies.

## Supplementary Information

ESM 1 (DOCX 2.23 MB)

## Abbreviations

CEM: Contrast-enhanced mammography
CE-MRI: Contrast-enhanced magnetic resonance imaging
CI: Confidence interval
DCIS: Ductal carcinoma in situ
IQR: Interquartile interval
PPV: Positive predictive value
rCEM: Recombined contrast-enhanced mammography images
SA: Standard assessment

## Declarations

### Guarantor

The scientific guarantor of this publication is Prof. Francesco Sardanelli, MD.

### Statistics and biometry

Andrea Cozzi and Giovanni Di Leo have significant statistical expertise.

### Study subjects or cohorts overlap

One-hundred twenty patients included in this study were previously reported in another article (doi: 10.3390/cancers14071774) that is however solely focused on the analysis of the radiation dose of CEM and does not involve any kind of consideration or analysis related to the topic of the present study.

### Methodology

• Prospective

• Diagnostic

• Multicentre
