# Supplementary material for: Contrast-enhanced mammography for the assessment of screening recalls: a two-centre study
Source: Eur Radiol. 2022 Jun 1;32(11):7388–99. doi: 10.1007/s00330-022-08868-3 (PMC9668944; doi:10.1007/s00330-022-08868-3)
Supplement: Supplementary file 1 — (DOCX 2.23 MB) [file 330_2022_8868_MOESM1_ESM.docx]

# Supplementary Methods

## Description of the screening program as implemented in the two study centres

Both centres in this study operate under the same regional regional legislative framework for both the spontaneous and organized breast cancer screening programs, the latter being managed by district-level Agencies for Health Protection controlled to the central Regional Healthcare System.

At both centres, women in the spontaneous screening group undergo annual two-view digital mammography upon their own request or after referral by a medical professional. Each mammography is evaluated by a single board-certified breast radiologist. Conversely, in the population-based organized screening program, biennial two-view digital mammography is offered to women aged 50–74 years, while annual two-view digital mammography is offered to women aged 45–49 years [1]. Each mammography is independently evaluated by two board-certified breast radiologists, according to the double-blind reading procedure [2]. In case of disagreement between the two readers, a review by consensus including a third reader is performed.

Under no circumstances screening with mammography plus ultrasound or screening with digital breast tomosynthesis is offered, neither in the spontaneous nor in the organized screening program.

All women recalled for suspicious findings at first level mammography, regardless of their screening group, are invited in the same centre of the first level examination to undergo imaging work-up (standard assessment), including breast ultrasound, digital breast tomosynthesis, and/or additional mammographic views (e.g., magnification, true lateral, etc.). Every week, a rotating breast radiologist performs the standard assessment on recalled women from both screening programs: thus, the radiologist who performs the second level evaluation does not necessarily correspond to the one who interpreted the first level mammography.

Enrollment in this study was offered to women aged 40–80 years (these cut-offs being determined for this study) recalled either from the spontaneous or from the organized breast cancer screening programs.
In this study, standard follow-up for all women from both the spontaneous and organized screening group consists in a two-view digital mammography performed after 2 years from the first level mammography, according to the standard screening interval proposed by our regional organized screening program. If a woman was enrolled from the spontaneous screening group or in the last round of her organized screening program, the follow-up digital mammography was offered within the study framework.

References

1. Schünemann HJ, Lerda D, Quinn C et al (2020) Breast Cancer Screening and Diagnosis: A Synopsis of the European Breast Guidelines. Ann Intern Med 172:46. doi:10.7326/M19-2125.

2. Perry N, Broeders M, de Wolf C, Törnberg S, Holland R, von Karsa L (2008) European guidelines for quality assurance in breast cancer screening and diagnosis. Fourth edition—summary document. Ann Oncol 19:614–622. doi:10.1093/annonc/mdm481.

## Table E1 Imaging features of the 90 lesions for which both standard assessment and contrast-enhanced mammography did not recommend a biopsy.

| **Patient ID / Lesion number** | **First level** | | **Second level** | | | | | | | | | **Third level** |
| --- | --- | --- | --- | --- | --- | --- | --- | --- | --- | --- | --- | --- |
|  | **Digital mammography** | | **Breast ultrasound** | | | | | **Digital mammographic magnification** | | **Digital breast tomosynthesis** | | **Biopsy** |
|  | Visibility | Features | Visibility | Echo pattern | Shape | Margins | Calcifications | Visibility | Calcifications | Visibility | Features | Result |
| 001.1 | Yes | Mass | No |  |  |  |  | – |  | No |  | – |
| 002.1 | Yes | Mass | No |  |  |  |  | – |  | No |  | – |
| 003.1 | Yes | Mass + Calc. | Yes | Anechoic | Round | Circumscribed | No | – |  | No |  | – |
| 006.1 | Yes | Mass | No |  |  |  |  | – |  | No |  | – |
| 007.1 | Yes | AD | No |  |  |  |  | – |  | No |  | – |
| 008.1 | Yes | Mass | No |  |  |  |  | – |  | No |  | – |
| 010.1 | Yes | Asymmetry | No |  |  |  |  | – |  | No |  | – |
| 012.1 | Yes | Mass | Yes | Anechoic | Oval | Circumscribed | No | – |  | – |  | – |
| 013.1 | Yes | Mass | Yes | Anechoic | Round | Circumscribed | No | – |  | No |  | – |
| 014.1 | Yes | Mass | No |  |  |  |  | – |  | No |  | – |
| 016.1 | Yes | Mass + Asymmetry | No |  |  |  |  | – |  | No |  | – |
| 019.1 | Yes | Mass | No |  |  |  |  | – |  | No |  | – |
| 020.1 | Yes | Calc. | No |  |  |  |  | Yes | Dystrophic | – |  | – |
| 022.1 | Yes | Mass | No |  |  |  |  | – |  | No |  | – |
| 023.1 | Yes | Mass | No |  |  |  |  | – |  | No |  | – |
| 026.1 | Yes | Mass | Yes | Anechoic | Round | Indistinct | No | – |  | – |  | – |
| 027.1 | Yes | Mass | No |  |  |  |  | – |  | No |  | – |
| 028.1 | Yes | Mass | No |  |  |  |  | – |  | No |  | – |
| 030.1 | Yes | Asymmetry | No |  |  |  |  | – |  | No |  | – |
| 031.1 | Yes | Mass | – |  |  |  |  | – |  | Yes | Mass | – |
| 033.1 | Yes | Mass | Yes | Anechoic | Round | Circumscribed | No | – |  | No |  | – |
| 037.1 | Yes | Mass | Yes | Anechoic | Round | Circumscribed | No | – |  | – |  | – |
| 037.2 | Yes | Mass | Yes ^a^ |  |  |  |  | – |  | – |  | – |
| 039.1 | Yes | Mass | No |  |  |  |  | – |  | No |  | – |
| 041.1 | Yes | Mass + Calc. | Yes | Hypoechoic | Irregular | Circumscribed | No | – |  | – |  | – |
| 042.1 | Yes | Mass | No |  |  |  |  | – |  | No |  | – |
| 043.1 | Yes | Mass + Calc. | Yes | Anechoic | Round | Circumscribed | No | – |  | – |  | – |
| 044.1 | Yes | Mass | No |  |  |  |  | – |  | No |  | – |
| 045.1 | Yes | Mass | No |  |  |  |  | – |  | No |  | – |
| 046.1 | Yes | Mass | No |  |  |  |  | – |  | No |  | – |
| 046.2 | Yes | Mass | No |  |  |  |  | – |  | No |  | – |
| 048.1 | Yes | Mass | Yes | Anechoic | Round | Circumscribed | No | – |  | Yes | Mass | – |
| 051.1 | Yes | Mass | No |  |  |  |  | – |  | No |  | – |
| 053.1 | Yes | Mass | No |  |  |  |  | – |  | No |  | – |
| 054.1 | Yes | Mass | No |  |  |  |  | – |  | No |  | – |
| 055.1 | Yes | Calc. | No |  |  |  |  | Yes | Amorphous | – |  | – |
| 057.1 | Yes | Calc. | Yes | Anechoic | Round | Circumscribed | No | Yes | Linear | – |  | – |
| 058.1 | Yes | Mass | No |  |  |  |  | – |  | No |  | – |
| 059.1 | Yes | Mass + Calc. | Yes ^b^ |  |  |  |  | – |  | No |  | – |
| 060.1 | Yes | Mass | No |  |  |  |  | – |  | No |  | – |
| 061.1 | Yes | Asymmetry | No |  |  |  |  | – |  | No |  | – |
| 062.1 | Yes | Mass | Yes | Anechoic | Oval | Indistinct | No | – |  | – |  | – |
| 063.1 | Yes | Mass + Calc. | Yes | Hypoechoic | Oval | Circumscribed | Yes | – |  | Yes | Mass + Calc. | – |
| 064.1 | Yes | Asymmetry | No |  |  |  |  | – |  | No |  | – |
| 066.1 | Yes | Mass | No |  |  |  |  | – |  | No |  | – |
| 068.1 | Yes | Calc. | No |  |  |  |  | Yes | Round | – |  | – |
| 070.1 | Yes | Mass | Yes | Anechoic | Round | Circumscribed | No | – |  | Yes | Mass | – |
| 072.1 | Yes | Mass + Asymmetry | No |  |  |  |  | – |  | No |  | – |
| 073.1 | Yes | Mass | Yes | Anechoic | Round | Circumscribed | No | – |  | Yes | Mass | – |
| 074.1 | Yes | Mass | No |  |  |  |  | – |  | No |  | – |
| 075.1 | Yes | Mass | No |  |  |  |  | – |  | Yes | Mass | – |
| 076.1 | Yes | Mass | Yes | Anechoic | Round | Circumscribed | No | – |  | No |  | – |
| 077.1 | Yes | Mass | Yes | Anechoic | Round | Indistinct | No | – |  | Yes | Mass | – |
| 078.1 | Yes | Mass | No |  |  |  |  | – |  | No |  | – |
| 079.1 | Yes | Mass | No |  |  |  |  | – |  | Yes | Mass | – |
| 081.1 | Yes | Mass + Calc. | No |  |  |  |  | – |  | – |  | – |
| 083.1 | Yes | Mass | Yes | Anechoic | Round | Circumscribed | No | – |  | Yes | Mass | – |
| 085.1 | Yes | Mass | Yes | Hyperechoic | Oval | Circumscribed | No | – |  | Yes | Mass | – |
| 087.1 | Yes | Calc. | Yes | Anechoic | Round | Circumscribed | Yes | Yes | Round | – |  | – |
| 088.1 | Yes | Mass | Yes | Anechoic | Round | Circumscribed | No | – |  | – |  | – |
| 091.1 | Yes | Mass | No |  |  |  |  | – |  | No |  | – |
| 095.1 | Yes | Asymmetry | No |  |  |  |  | – |  | No |  | – |
| 096.1 | Yes | Mass + AD | No |  |  |  |  | – |  | No |  | – |
| 098.1 | Yes | Calc. | No |  |  |  |  | Yes | Dystrophic | – |  | – |
| 101.1 | Yes | Mass | No |  |  |  |  | – |  | No |  | – |
| 105.1 | Yes | Mass | – |  |  |  |  | – |  | No |  | – |
| 107.1 | Yes | Mass | Yes | Anechoic | Oval | Circumscribed | No | – |  | – |  | – |
| 108.1 | Yes | Mass + Calc. | Yes | Anechoic | Round | Circumscribed | No | – |  | – |  | – |
| 112.1 | Yes | Mass + AD | No |  |  |  |  | – |  | – |  | – |
| 114.1 | Yes | Mass | No |  |  |  |  | – |  | No |  | – |
| 114.2 | Yes | Calc. | No |  |  |  |  | Yes | Coarse heterogeneous | – |  | – |
| 115.1 | Yes | Mass + Calc. | Yes | Hypoechoic | Oval | Circumscribed | Yes | – |  | – |  | – |
| 116.1 | Yes | Mass | No |  |  |  |  | – |  | – |  | – |
| 117.1 | Yes | Mass | Yes | Hypoechoic | Round | Indistinct | No | – |  | No |  | – |
| 119.1 | Yes | Mass + Asymmetry | No |  |  |  |  | – |  | No |  | – |
| 120.1 | Yes | AD | No |  |  |  |  | – |  | No |  | – |
| 122.1 | Yes | Mass | No |  |  |  |  | – |  | No |  | – |
| 123.1 | Yes | Mass | Yes | Anechoic | Round | Circumscribed | No | – |  | Yes | Mass | – |
| 124.1 | Yes | Calc. | Yes | Anechoic | Round | Circumscribed | No | No |  | – |  | – |
| 125.1 | Yes | Mass + Asymmetry | No |  |  |  |  | – |  | No |  | – |
| 135.1 | Yes | Mass | No |  |  |  |  | – |  | No |  | – |
| 144.1 | Yes | Calc. | No |  |  |  |  | No |  | – |  | – |
| 148.2 | Yes | Mass | Yes | Isoechoic | Oval | Indistinct | No | – |  | Yes | Mass | – |
| 160.1 | Yes | Asymmetry | No |  |  |  |  | – |  | No |  | – |
| 183.1 | Yes | Mass | No |  |  |  |  | – |  | No |  | – |
| 184.1 | Yes | Mass | Yes | Anechoic | Round | Circumscribed | No | – |  | Yes | Mass | – |
| 187.1 | Yes | Calc. | No |  |  |  |  | No |  | – |  | – |
| 192.1 | Yes | Asymmetry | No |  |  |  |  | No |  | – |  | – |
| 198.1 | Yes | Mass | Yes | Anechoic | Round | Circumscribed | No | – |  | Yes | Mass | – |
| 217.1 | Yes | Mass + Asymmetry | No |  |  |  |  | – |  | Yes | Mass | – |

All imaging features are reported according to the BI-RADS lexicon for each modality. Dashes under the “Visibility” columns indicate that the examination was not performed.

*Calc.* calcifications; *AD* architectural distortion.

^a^ Enlarged intramammary lymph node with normal circumscribed oval appearance, without calcifications.

^b^ Hypoechoic area consistent with post-surgical breast scar.

## Table E2 Imaging features and biopsy results of the 90 lesions for which biopsy was recommended both by standard assessment and contrast-enhanced mammography.

| **Patient ID / Lesion number** | **First level** | | **Second level** | | | | | | | | | **Third level** |
| --- | --- | --- | --- | --- | --- | --- | --- | --- | --- | --- | --- | --- |
|  | **Digital mammography** | | **Breast ultrasound** | | | | | **Digital mammographic magnification** | | **Digital breast tomosynthesis** | | **Biopsy** |
|  | Visibility | Features | Visibility | Echo pattern | Shape | Margins | Calcifications | Visibility | Calcifications | Visibility | Features | Result |
| 38.1 | Yes | Mass | Yes | Hypoechoic | Irregular | Microlobulated | No | – |  | – |  | B1 |
| 213.1 | Yes | Asymmetry | Yes | Anechoic | Round | Circumscribed | No | – |  | Yes | Mass | B1 |
| 15.1 | Yes | Mass + Calc. | No |  |  |  |  | Yes | Amorphous | – |  | B2 |
| 36.1 | Yes | Mass | Yes | Hypoechoic | Irregular | Indistinct | No | – |  | – |  | B2 |
| 40.1 | Yes | Mass | Yes | Hypoechoic | Irregular | Circumscribed | No | – |  | Yes | Mass | B2 |
| 50.1 | Yes | Mass | Yes | Hypoechoic | Oval | Indistinct | No | – |  | Yes | Mass | B2 |
| 50.2 | No ^a^ |  | Yes | Hyperechoic | Oval | Indistinct | No | – |  | Yes | Mass | B2 |
| 93.1 | Yes | Mass + Calc. | No |  |  |  |  | – |  | – |  | B2 |
| 97.1 | Yes | Calc. | Yes | Heterogeneous | Round | Indistinct | Yes | – |  | – |  | B2 |
| 109.1 | Yes | Mass + Calc. | No |  |  |  |  | Yes | Round | – |  | B2 |
| 143.1 | Yes | Calc. | – |  |  |  |  | Yes | Amorphous | – |  | B2 |
| 150.1 | Yes | Mass | Yes | Hypoechoic | Oval | Circumscribed | No | – |  | Yes | Mass | B2 |
| 206.2 | No ^a^ |  | Yes | Hypoechoic | Round | Indistinct | No | – |  | No |  | B2 |
| 149.1 | Yes | Mass | Yes | Hypoechoic | Oval | Circumscribed | No | – |  | – |  | C2 |
| 149.2 | No ^a^ |  | Yes | Hypoechoic | Oval | Indistinct | No | – |  | – |  | C2 |
| 197.1 | Yes | Calc. | No |  |  |  |  | Yes | Round | No |  | B3 → FU |
| 161.1 | Yes | Calc. | – |  |  |  |  | – |  | – |  | B3 → B5b |
| 113.1 | Yes | Mass + Calc. | Yes | Hypoechoic | Irregular | Indistinct | No | – |  | No |  | B5a |
| 146.1 | Yes | Calc. | – |  |  |  |  | – |  | – |  | B5a |
| 156.1 | Yes | Calc. | – |  |  |  |  | – |  | – |  | B5a |
| 158.1 | Yes | Calc. | – |  |  |  |  | – |  | – |  | B5a |
| 162.1 | Yes | Calc. | Yes | Heterogeneous | Irregular | Indistinct | Yes | – |  | – |  | B5a |
| 165.1 | Yes | Calc. | Yes | Hypoechoic | Irregular | Indistinct | Yes | – |  | – |  | B5a |
| 168.1 | Yes | Calc. | – |  |  |  |  | – |  | – |  | B5a |
| 185.1 | Yes | Calc. | – |  |  |  |  | – |  | No |  | B5a |
| 186.1 | Yes | Calc. | No | Complex cystic and solid | Round | Circumscribed | No | – |  | – |  | B5a |
| 190.1 | Yes | Calc. | Yes | Hypoechoic | Irregular | Indistinct | Yes | – |  | – |  | B5a |
| 193.1 | Yes | Mass + Calc. | Yes | Hypoechoic | Irregular | Indistinct | No | – |  | – |  | B5a |
| 208.1 | Yes | Calc. | No |  |  |  |  | Yes | Pleomorphic | – |  | B5a |
| 18.1 | Yes | Asymmetry | Yes | Hypoechoic | Irregular | Indistinct | No | – |  | – |  | B5b |
| 25.1 | Yes | Calc. | Yes | Hypoechoic | Irregular | Indistinct | No | Yes | Pleomorphic | – |  | B5b |
| 29.1 | Yes | Mass | Yes | Hypoechoic | Irregular | Indistinct | No | – |  | Yes | Mass | B5b |
| 35.1 | Yes | Mass | Yes | Hypoechoic | Irregular | Indistinct | No | – |  | Yes | Mass | B5b |
| 47.1 | Yes | Mass | Yes | Hypoechoic | Irregular | Spiculated | No | – |  | Yes | Mass | B5b |
| 56.1 | Yes | Mass | Yes | Hypoechoic | Irregular | Indistinct | No | – |  | Yes | Mass | B5b |
| 65.1 | Yes | Mass | Yes | Hypoechoic | Irregular | Indistinct | No | – |  | Yes | Mass | B5b |
| 67.1 | Yes | Mass + Calc. | Yes | Hypoechoic | Round | Indistinct | No | – |  | Yes | Mass + Calc. | B5b |
| 80.1 | Yes | Mass | Yes | Hypoechoic | Irregular | Indistinct | No | – |  | Yes | Mass | B5b |
| 80.2 | Yes | Mass | Yes ^b^ |  |  |  |  | – |  | Yes | Mass | B5b |
| 84.1 | Yes | Mass + Calc. + Asymmetry | Yes | Hypoechoic | Irregular | Indistinct | No | – |  | Yes | Mass + Calc. | B5b |
| 89.1 | Yes | Mass | Yes | Hyperechoic | Oval | Circumscribed | No | – |  | No |  | B5b |
| 100.1 | Yes | Mass | Yes | Hypoechoic | Irregular | Circumscribed | No | – |  | Yes | Mass | B5b |
| 104.1 | Yes | Calc. | No |  |  |  |  | Yes | Linear | – |  | B5b |
| 127.1 | Yes | Mass | Yes | Hypoechoic | Irregular | Indistinct | No | – |  | Yes | Mass | B5b |
| 127.2 | Yes | Mass | Yes | Hypoechoic | Round | Indistinct | No | – |  | Yes | Mass | B5b |
| 128.1 | Yes | Calc. | No |  |  |  |  | – |  | Yes | Calc. | B5b |
| 128.2 | Yes | Mass + AD | Yes | Hypoechoic | Irregular | Indistinct | No | – |  | Yes | Mass + AD | B5b |
| 130.1 | Yes | Calc. | – |  |  |  |  | – |  | – |  | B5b |
| 133.1 | Yes | Calc. | Yes |  |  |  | Yes | – |  | – |  | B5b |
| 133.2 | Yes | Calc. + Asymmetry | No |  |  |  |  | Yes | Linear | – |  | B5b |
| 137.1 | Yes | Mass + Calc. | Yes | Hypoechoic | Irregular | Indistinct | Yes | – |  | Yes | Mass + Calc. | B5b |
| 138.1 | Yes | Calc. + AD | Yes | Hypoechoic | Irregular | Indistinct | No | – |  | Yes | Calc. + AD | B5b |
| 147.1 | Yes | Mass | Yes | Hypoechoic | Irregular | Indistinct | No | – |  | Yes | Mass | B5b |
| 148.1 | Yes | AD | Yes | Hypoechoic | Irregular | Indistinct | No | – |  | Yes | AD | B5b |
| 151.1 | Yes | Mass | Yes | Hypoechoic | Irregular | Indistinct | No | – |  | – |  | B5b |
| 154.1 | Yes | Mass + Asymmetry | Yes | Hypoechoic | Irregular | Indistinct | No | – |  | Yes | Mass | B5b |
| 155.1 | Yes | Mass + Calc. | Yes | Hypoechoic | Round | Indistinct | No | – |  | – |  | B5b |
| 157.1 | Yes | Calc. | No | Anechoic | Round | Circumscribed | No | – |  | – |  | B5b |
| 159.1 | Yes | Calc. | No |  |  |  |  | – |  | Yes | Calc. | B5b |
| 163.1 | Yes | Mass + AD | Yes | Hypoechoic | Irregular | Indistinct | No | – |  | Yes | Calc. | B5b |
| 164.1 | Yes | Calc. | No |  |  |  |  | Yes | Coarse heterogeneous | – |  | B5b |
| 166.1 | Yes | Mass + AD | Yes | Hypoechoic | Irregular | Indistinct | No | – |  | Yes | Mass + AD | B5b |
| 170.1 | Yes | Mass | Yes | Hypoechoic | Irregular | Indistinct | No | – |  | No |  | B5b |
| 171.1 | Yes | Mass + AD | Yes | Hypoechoic | Irregular | Indistinct | No | – |  | Yes | Mass + AD | B5b |
| 174.1 | Yes | Calc. + Asymmetry | – |  |  |  |  | – |  | – |  | B5b |
| 176.1 | Yes | Mass | Yes | Hypoechoic | Irregular | Indistinct | No | – |  | Yes | Mass | B5b |
| 177.1 | Yes | Calc. | – |  |  |  |  | Yes | Linear | – |  | B5b |
| 178.1 | Yes | Mass | Yes | Hypoechoic | Irregular | Indistinct | No | – |  | Yes | Mass | B5b |
| 179.1 | Yes | Mass | Yes | Hypoechoic | Irregular | Circumscribed | No | – |  | Yes | Mass | B5b |
| 181.1 | Yes | Mass + Calc. + AD | Yes | Hypoechoic | Irregular | Indistinct | No | – |  | Yes | Mass + AD | B5b |
| 191.1 | Yes | AD | No |  |  |  |  | – |  | Yes | AD | B5b |
| 194.1 | Yes | Mass + Calc. | Yes | Hypoechoic | Irregular | Indistinct | Yes | – |  | Yes | Mass + Calc. | B5b |
| 195.1 | Yes | Mass | Yes | Hypoechoic | Irregular | Spiculated | No | – |  | Yes | Mass | B5b |
| 196.1 | Yes | Calc. | – |  |  |  |  | – |  | – |  | B5b |
| 200.1 | Yes | Mass | Yes | Hypoechoic | Round | Indistinct | No | – |  | Yes | Mass | B5b |
| 201.1 | Yes | Mass + AD | Yes | Hypoechoic | Irregular | Spiculated | No | – |  | Yes | Mass + AD | B5b |
| 202.1 | Yes | AD | No |  |  |  |  | – |  | Yes | AD | B5b |
| 203.1 | Yes | Mass | Yes | Hypoechoic | Round | Indistinct | No | – |  | Yes | Mass | B5b |
| 204.1 | Yes | AD | No |  |  |  |  | – |  | No |  | B5b |
| 205.1 | Yes | Mass | Yes | Hypoechoic | Irregular | Spiculated | No | – |  | Yes | Mass | B5b |
| 209.1 | Yes | Mass + Asymmetry | Yes | Hypoechoic | Irregular | Spiculated | No | – |  | Yes | Mass | B5b |
| 210.1 | Yes | Asymmetry | Yes | Hypoechoic | Oval | Indistinct | No | – |  | Yes | Mass | B5b |
| 211.1 | Yes | Calc. + AD | Yes | Hypoechoic | Irregular | Indistinct | No | – |  | Yes | Calc. + AD | B5b |
| 212.1 | Yes | Mass + Calc. | Yes | Hypoechoic | Irregular | Indistinct | No | – |  | – |  | B5b |
| 216.1 | Yes | AD | No |  |  |  |  | Yes | Pleomorphic | Yes | AD | B5b |
| 219.1 | Yes | Mass | Yes | Hypoechoic | Irregular | Indistinct | No | – |  | Yes | Mass | B5b |
| 18.2 | Yes | Mass | Yes ^b^ |  |  |  |  | – |  | – |  | B5d |
| 25.2 | No ^a^ |  | Yes ^b^ |  |  |  |  | – |  | – |  | B5d |
| 154.2 | Yes | Mass | Yes ^b^ |  |  |  |  | – |  | Yes | Mass | B5d |
| 106.1 | Yes | Calc. | – |  |  |  |  | Yes | Pleomorphic | – |  | Unknown ^c^ |

All imaging features are reported according to the BI-RADS lexicon for each modality. Dashes under the “Visibility” columns indicate that the examination was not performed.
*Calc.* calcifications; *FU* follow-up; *AD* architectural distortion.

^a^ Ultrasound detected additional finding.

^b^ Enlarged axillary lymph node with thickened cortices, consistent with metastatic lymph node.

^c^ The patient elected to undergo biopsy in another centre and was lost to follow-up.

## Table E3 Imaging features and biopsy results of the 41 lesions for which biopsy was recommended by standard assessment but not by contrast-enhanced mammography.

| **Patient ID / Lesion number** | **First level** | | **Second level** | | | | | | | | | **Third level** |
| --- | --- | --- | --- | --- | --- | --- | --- | --- | --- | --- | --- | --- |
|  | **Digital mammography** | | **Breast ultrasound** | | | | | **Digital mammographic magnification** | | **Digital breast tomosynthesis** | | **Biopsy** |
|  | Visibility | Features | Visibility | Echo pattern | Shape | Margins | Calcifications | Visibility | Calcifications | Visibility | Features | Result |
| 4.1 | Yes | Mass | Yes | Hypoechoic | Round | Indistinct | No | – |  | No |  | B2 |
| 5.1 | No ^a^ |  | Yes | Hypoechoic | Oval | Circumscribed | No | – |  | No |  | B2 |
| 32.1 | Yes | Mass | No |  |  |  |  | – |  | Yes | Mass | B2 |
| 34.1 | Yes | Calc. | No |  |  |  |  | Yes | Dystrophic | – |  | B2 |
| 86.1 | No ^b^ |  | Yes | Hypoechoic | Round | Indistinct | No | – |  | No |  | B2 |
| 90.1 | Yes | Mass | Yes | Hypoechoic | Round | Indistinct | No | – |  | Yes | Mass | B2 |
| 92.1 | Yes | Calc. | No |  |  |  |  | Yes | Round | – |  | B2 |
| 94.1 | Yes | Mass | No |  |  |  |  | – |  | Yes | Mass | B2 |
| 99.1 | Yes | Calc. | No |  |  |  |  | Yes | Amorphous | – |  | B2 |
| 102.1 | Yes | Mass | Yes | Hypoechoic | Round | Indistinct | No | – |  | – |  | B2 |
| 110.1 | Yes | Mass + Calc. | Yes | Hypoechoic | Irregular | Circumscribed | No | – |  | Yes | Mass + Calc. | B2 |
| 118.1 | Yes | Calc. | No |  |  |  |  | Yes | Amorphous | – |  | B2 |
| 121.1 | Yes | Mass | Yes | Hypoechoic | Oval | Indistinct | No | – |  | Yes | Mass | B2 |
| 132.1 | Yes | Calc. | Yes | Heterogeneous | Irregular | Indistinct | Yes | – |  | – |  | B2 |
| 153.1 | Yes | Calc. | – |  |  |  |  | – |  | – |  | B2 |
| 167.1 | Yes | Calc. | No |  |  |  |  | – |  | – |  | B2 |
| 169.1 | Yes | Calc. | No |  |  |  |  | Yes | Amorphous | – |  | B2 |
| 172.1 | Yes | Calc. | – |  |  |  |  | – |  | – |  | B2 |
| 188.1 | Yes | Calc. | Yes | Hypoechoic | Irregular | Indistinct | No | Yes | Amorphous | – |  | B2 |
| 189.1 | Yes | Mass + Asymmetry | Yes | Heterogeneous | Irregular | Circumscribed | No | – |  | Yes | Mass | B2 |
| 199.1 | Yes | Calc. | – |  |  |  |  | – |  | – |  | B2 |
| 206.1 | Yes | Calc. | Yes | Hypoechoic | Round | Indistinct | Yes | No |  | No |  | B2 |
| 215.1 | Yes | Calc. | No |  |  |  |  | – |  | – |  | B2 |
| 220.1 | Yes | AD | Yes | Hypoechoic | Irregular | Spiculated | No | – |  | Yes | AD | B2 |
| 82.1 | Yes | Mass | Yes | Anechoic | Round | Indistinct | No | – |  | Yes | Mass | C2 |
| 137.2 | No ^c^ |  | Yes ^d^ |  |  |  |  | – |  | – |  | C2 |
| 195.2 | No ^c^ |  | Yes ^e^ |  |  |  |  | – |  | – |  | C2 |
| 129.1 | Yes | Calc. | – |  |  |  |  | – |  | – |  | B3 → FU |
| 141.1 | Yes | Calc. | – |  |  |  |  | – |  | – |  | B3 → FU |
| 180.1 | No ^f^ |  | Yes | Heterogeneous | Irregular | Circumscribed | No | – |  | – |  | B3 → FU |
| 131.1 | Yes | Calc. | No |  |  |  |  | – |  | – |  | B3 → B2 |
| 134.1 | Yes | Calc. | No |  |  |  |  | – |  | Yes | Calc. | B3 → B2 |
| 21.1 | Yes | Calc. | No |  |  |  |  | Yes | Pleomorphic | – |  | B5a |
| 52.1 | Yes | Calc. | No |  |  |  |  | Yes | Amorphous | – |  | B5a |
| 140.1 | Yes | Calc. | – |  |  |  |  | Yes | Amorphous | – |  | B5a |
| 145.1 | Yes | Calc. | – |  |  |  |  | – |  | – |  | B5a |
| 207.1 | Yes | Calc. | Yes | Anechoic | Round | Circumscribed | Yes | – |  | – |  | B5a |
| 139.1 | Yes | Calc. | No |  |  |  |  | – |  | – |  | Unfeasible |
| 173.1 | Yes | Calc. | No |  |  |  |  | No |  | – |  | Unfeasible |
| 214.1 | Yes | Calc. | No |  |  |  |  | – |  | – |  | Unfeasible |
| 111.1 | Yes | Calc. | No |  |  |  |  | – |  | – |  | Unknown ^g^ |

All imaging features are reported according to the BI-RADS lexicon for each modality. Dashes under the “Visibility” columns indicate that the examination was not performed.

*Calc.* calcifications; *AD* architectural distortion; *FU* follow-up.

^a^ Ultrasound detected suspicious lesion in the lower-outer quadrant of the right breast in a woman recalled for a mammographic suspicious finding in the contralateral breast.

^b^ Ultrasound detected suspicious lesion in the upper-inner of the right breast in a woman recalled for a mammographic suspicious finding in the outer quadrants of the ipsilateral breast.

^c^ Ultrasound detected additional finding

^d^ Enlarged axillary lymph node with thickened cortices, consistent with metastatic lymph node.

^e^ Enlarged axillary lymph node, probably reactive.

^f^ Ultrasound detected suspicious lesion in the outer quadrants of the left breast in a woman recalled for a mammographic suspicious finding in the contralateral breast.

^g^ The patient elected to undergo biopsy in another centre and was lost to follow-up.

## Table E4 Imaging features and biopsy results of the 4 lesions for which a biopsy was recommended only by contrast-enhanced mammography, after standard assessment had either recommended a referral to follow-up or had not identified the lesion.

| **Patient ID / Lesion number** | **First level** | | **Second level** | | | | | | | | | **Third level** |
| --- | --- | --- | --- | --- | --- | --- | --- | --- | --- | --- | --- | --- |
|  | **Digital mammography** | | **Breast ultrasound** | | | | | **Digital mammographic magnification** | | **Digital breast tomosynthesis** | | **Biopsy** |
|  | Visibility | Features | Visibility | Echo pattern | Shape | Margins | Calcifications | Visibility | Calcifications | Visibility | Features | Result |
| 104.2 | No ^a^ |  | No |  |  |  |  | – |  | – |  | B5a |
| 011.1 | Yes | Calc. | No |  |  |  |  | Yes | Linear | – |  | B5b |
| 024.1 | Yes | Calc. | – |  |  |  |  | Yes | Amorphous | No |  | Unknown ^b^ |
| 026.2 | No ^a^ |  | – |  |  |  |  | – |  | – |  | Unknown ^b^ |

All imaging features are reported according to the BI-RADS lexicon for each modality. Dashes under the “Visibility” columns indicate that the examination was not performed.

*Calc.* calcifications.

^a^ This finding was identified only by contrast uptake at contrast-enhanced mammography.

^b^ The patient refused to undergo the biopsy recommended by contrast-enhanced mammography in contrast to the follow-up recommended by standard assessment.

# Supplementary figures

**
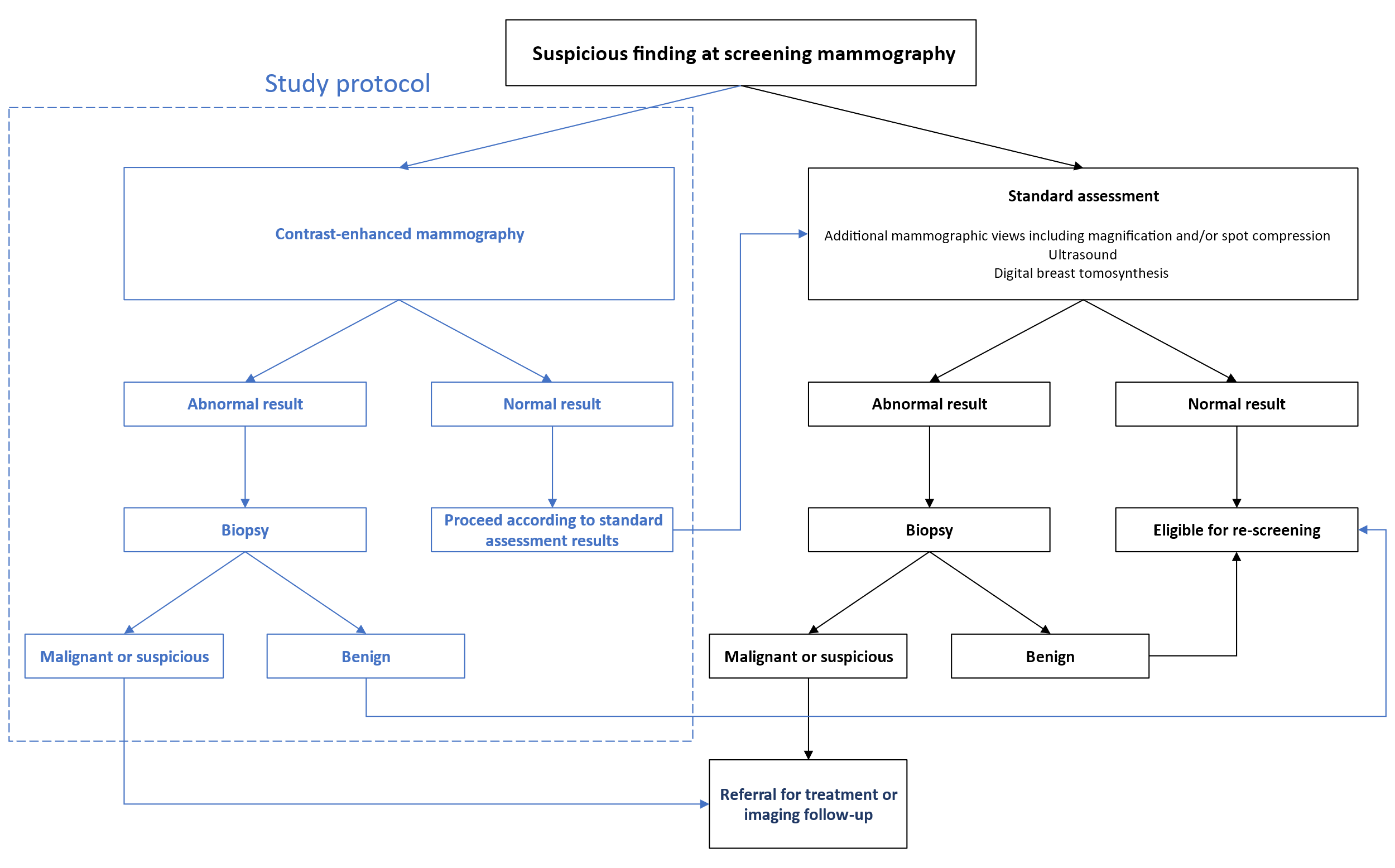
Fig. E1** Flowchart of the standard work-up process in the two study centres supplemented by contrast-enhanced mammography, as per study protocol.


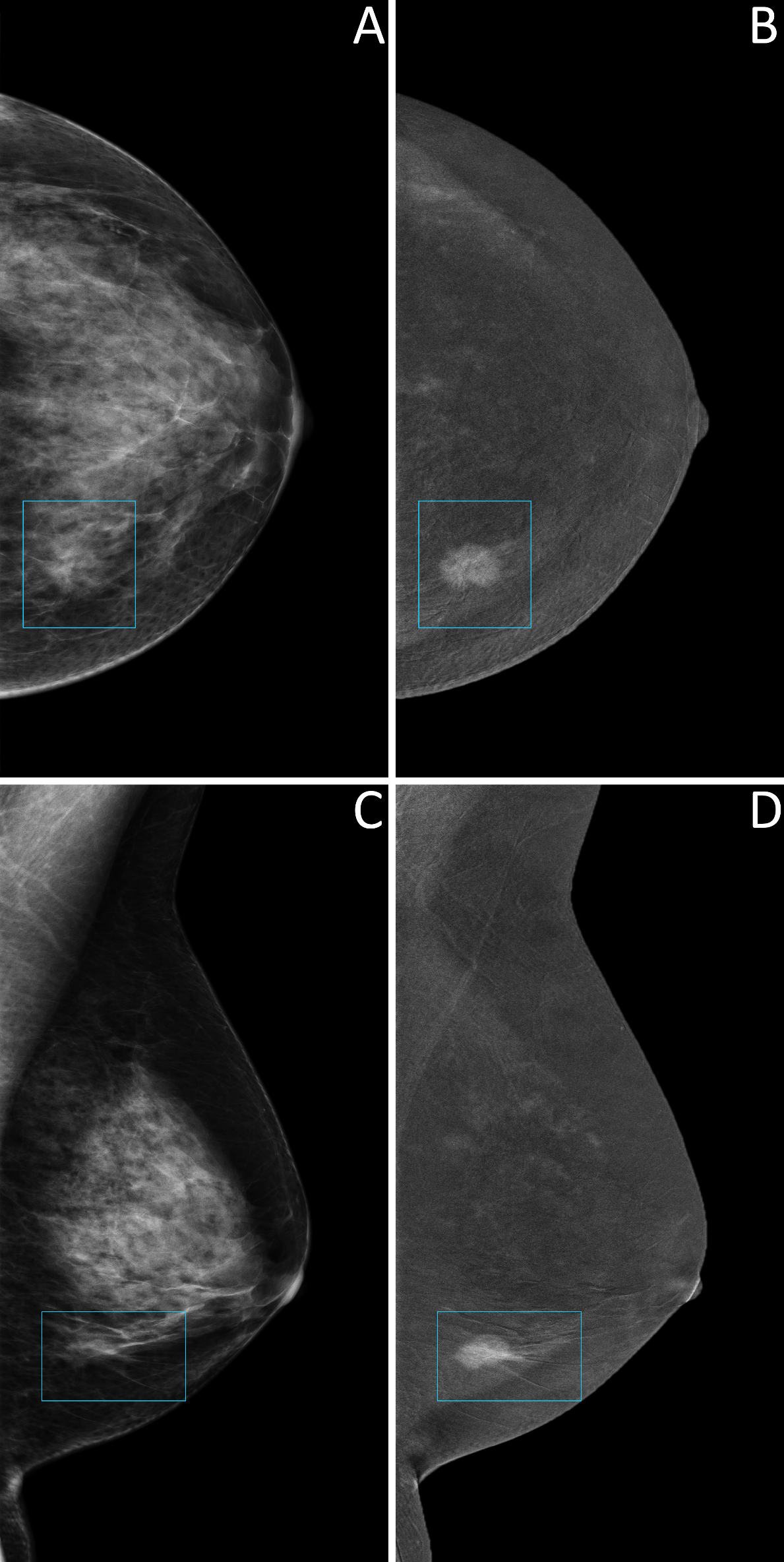
**Fig. E2**

True positive case at contrast-enhanced mammography. A 61-year-old woman was recalled for a suspicious asymmetric opacity in the lower-inner quadrant of the left breast, also recognizable on low-energy images (panels A and C, light blue rectangles) and subsequently diagnosed as an invasive carcinoma of no special type, grade 1. Recombined images (panels B and D, light blue rectangles) show an oval enhancing and irregularly-shaped mass of 14 mm.
